# Comparison of MRI and VQ-SPECT as a Screening Test for Patients With Suspected CTEPH: CHANGE-MRI Study Design and Rationale

**DOI:** 10.3389/fcvm.2020.00051

**Published:** 2020-04-09

**Authors:** Florian Lasch, Annika Karch, Armin Koch, Thorsten Derlin, Andreas Voskrebenzev, Tawfik Moher Alsady, Marius M. Hoeper, Henning Gall, Fritz Roller, Sebastian Harth, Dagmar Steiner, Gabriele Krombach, Hossein Ardeschir Ghofrani, Fabian Rengier, Claus Peter Heußel, Ekkehard Grünig, Dietrich Beitzke, Marcus Hacker, Irene M. Lang, Jürgen Behr, Peter Bartenstein, Julien Dinkel, Kai-Helge Schmidt, Karl-Friedrich Kreitner, Thomas Frauenfelder, Silvia Ulrich, Okka W. Hamer, Michael Pfeifer, Christopher S. Johns, David G. Kiely, Andrew James Swift, Jim Wild, Jens Vogel-Claussen

**Affiliations:** ^1^Institute of Biostatistics, Hannover Medical School, Hanover, Germany; ^2^Department of Nuclear Medicine, Hannover Medical School, Hanover, Germany; ^3^Department of Diagnostic and Interventional Radiology, Hannover Medical School, Hanover, Germany; ^4^Department of Pneumology, Hannover Medical School, Hanover, Germany; ^5^Biomedical Research in Endstage and Obstructive Lung Disease Hannover (BREATH), Member of the German Center for Lung Research (DZL), Hanover, Germany; ^6^Department of Pneumology, Medical University Giessen and Marburg, Giessen, Germany; ^7^Universities of Giessen and Marburg Lung Center, Marburg, Germany; ^8^Department of Radiology, Medical University Giessen and Marburg, Giessen, Germany; ^9^Department of Nuclear Medicine, Medical University Giessen and Marburg, Giessen, Germany; ^10^Diagnostic and Interventional Radiology, University Hospital Heidelberg, Heidelberg, Germany; ^11^Translational Lung Research Center (TLRC) Heidelberg, Member of the German Center for Lung Research (DZL), University of Heidelberg, Heidelberg, Germany; ^12^Department of Diagnostic and Interventional Radiology With Nuclear Medicine, Thoraxklinik at University Hospital Heidelberg, Heidelberg, Germany; ^13^Centre for Pulmonary Hypertension, Thoraxklinik at Heidelberg University Hospital, Heidelberg, Germany; ^14^Department of Biomedical Imaging and Image-Guided Therapy, Medical University of Vienna, Vienna, Austria; ^15^Department of Cardiology, Internal Medicine II, Medical University of Vienna, Vienna, Austria; ^16^Department of Pneumology, Ludwig-Maximilan University Munich, Munich, Germany; ^17^Comprehensive Pneumology Center, Munich, Ludwig-Maximilians-University Munich and Asklepios Hospital Munich-Gauting and Helmholtz Center Munich, Member of the German Center for Lung Research (DZL), Munich, Germany; ^18^Department of Nuclear Medicine, Ludwig-Maximilan University, Munich, Germany; ^19^Department of Radiology, Ludwig-Maximilan University Munich, Munich, Germany; ^20^Department of Cardiology, University Medical Center of the Johannes Gutenberg University Mainz, Mainz, Germany; ^21^Department of Diagnostic and Interventional Radiology, University Medical Center of the Johannes Gutenberg University Mainz, Mainz, Germany; ^22^Department of Diagnostic and Interventional Radiology, University Hospital Zurich, Zurich, Switzerland; ^23^Department of Respiratory Medicine, Pulmonary Hypertension Unit, University Hospital Zurich, Zurich, Switzerland; ^24^Department of Radiology, Regensburg University Hospital, Regensburg, Germany; ^25^Department of Pneumology, Regensburg University Hospital, Regensburg, Germany; ^26^Academic Unit of Radiology, University of Sheffield, Sheffield, United Kingdom; ^27^Sheffield Pulmonary Vascular Disease Unit, Royal Hallamhire Hospital, Sheffield, United Kingdom; ^28^Department of Infection, Immunity and Cardiovascular Disease, University of Sheffield, Sheffield, United Kingdom

**Keywords:** MRI, VQ-SPECT, CTEPH, PH, pulmonary embolism, diagnostic strategy

## Abstract

The diagnostic strategy for chronic thromboembolic pulmonary hypertension (CTEPH) is composed of two components required for a diagnosis of CTEPH: the presence of chronic pulmonary embolism and an elevated pulmonary artery pressure. The current guidelines require that ventilation–perfusion single-photon emission computed tomography (VQ-SPECT) is used for the first step diagnosis of chronic pulmonary embolism. However, VQ-SPECT exposes patients to ionizing radiation in a radiation sensitive population. The prospective, multicenter, comparative phase III diagnostic trial **C**TEP**H** di**agn**osis **E**urope - MRI (CHANGE-MRI, ClinicalTrials.gov identifier *NCT02791282*) aims to demonstrate whether functional lung MRI can serve as an equal rights alternative to VQ-SPECT in a diagnostic strategy for patients with suspected CTEPH. Positive findings are verified with catheter pulmonary angiography or computed tomography pulmonary angiography (gold standard). For comparing the imaging methods, a co-primary endpoint is used. (i) the proportion of patients with positive MRI in the group of patients who have a positive SPECT and gold standard diagnosis for chronic pulmonary embolism and (ii) the proportion of patients with positive MRI in the group of patients with negative SPECT and gold standard. The CHANGE-MRI trial will also investigate the performance of functional lung MRI without i.v. contrast agent as an index test and identify cardiac, hemodynamic, and pulmonary MRI-derived parameters to estimate pulmonary artery pressures and predict 6–12 month survival. Ultimately, this study will provide the necessary evidence for the discussion about changes in the recommendations on the diagnostic approach to CTEPH.

## Introduction

### The Clinical Problem

**Figure 1:**
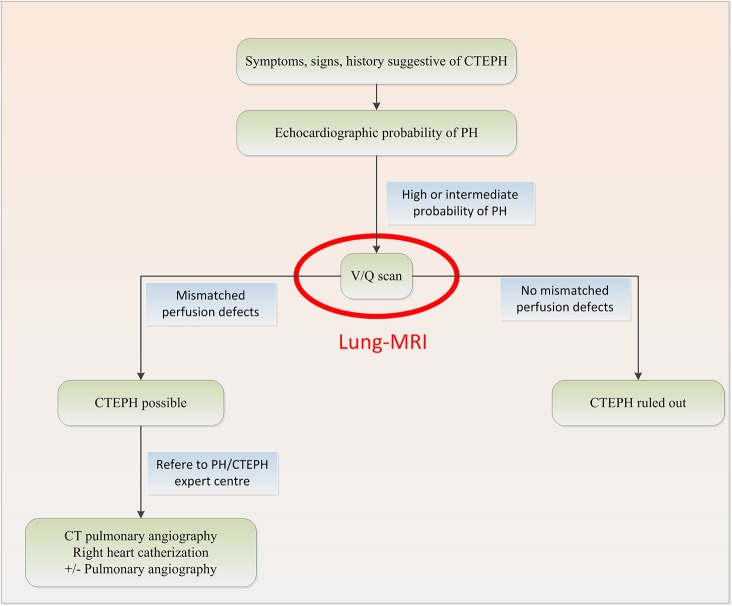
Diagnostic algorithm for CTEPH based on the 2015 ESC/ERS Guidelines for the diagnosis and treatment of pulmonary hypertension with potential change in red (7, 8).

Chronic thromboembolic pulmonary hypertension (CTEPH) occurs in 0.1–4.0% of patients with acute pulmonary embolism (PE) within 2 years (1). If left untreated, the outlook for patients with CTEPH is dismal. Median survival is <2 years in patients who have a mean pulmonary artery pressure higher than 30 mm Hg at diagnosis (2). Right-heart failure is the most frequent cause of death. However, when treated successfully with endarterectomy, CTEPH patients have a good prognostic outcome (3), therefore timely diagnosis is of great importance. Advances in management have improved outcomes, but CTEPH remains a potentially fatal condition, especially when surgery is not an option (4, 5). The diagnostic approach according to the current guidelines for CTEPH starts with transthoracic echocardiography (6) to assess the likelihood of pulmonary hypertension, followed by ventilation-perfusion single-photon emission computed tomography (VQ-SPECT) to detect or rule out perfusion defects (7) (Figure 1). VQ-SPECT is currently the preferred imaging tool for screening because of its high sensitivity and a negative predictive value of virtually 100% (9).

### The Need for a Trial

**Figure 2:**
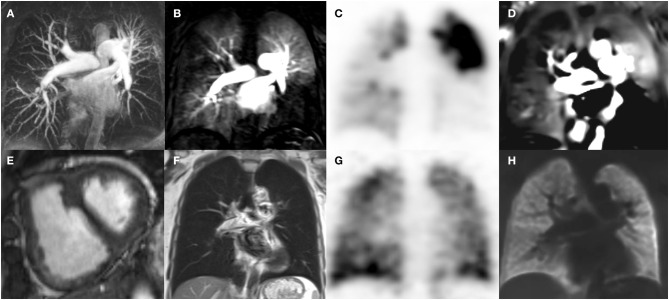
Cardio-pulmonary MRI **(A,B,D,E,F,H)** and V/Q SPECT **(C,G)** images of a patient with CTEPH. MRI angiography [coronal maximum intensity projection, **(A)**] Maximum intensity projection (MIP) depicts pulmonary artery stenosis and irregularities predominantly in the lower lobes as well as in the left upper lobe. Time resolved 4D MRI perfusion DCE angiography (coronal, acquisition time per volume: 1.3 s, voxel size 3 × 4 × 5 mm, **B**) depicts corresponding parenchymal hypoperfusion, which, matches with the Q-SPECT (coronal, voxel size 5 × 5 × 12 mm, **C**) findings. V-SPECT (coronal, voxel size 5 × 5 × 12 mm, **G**) shows relatively homogeneous ventilation. In accordance, neither infiltrates nor atelectasis are seen on T2 weighted Half-Fourier Acquisition Single-shot Turbo spin Echo (HASTE)-MRI (coronal, voxel size 2 × 2 × 6 mm, **F**), notice the signal in the main pulmonary arteries due to slow flow. V/Q Phase resolved functional lung MRI (PREFUL, coronal, voxel size 4 × 4 × 15 mm, **D,H**) depicts normal regional ventilation **(H)** and regions of hypoperfusion matching the V/Q SPECT **(C,G)** and DCE MRI **(B)** findings. Short axis cardiac cine MRI shows right ventricular hypertrophy and septal flattening (arrow) in systole due to increased pulmonary vascular resistance.

VQ-SPECT requires ionizing radiation, while MRI systems are not associated with any additional radiation dose and have increasing availability. Initially, a multicenter study conducted between 2006 and 2008 in 371 patients with suspected pulmonary embolism using Gadolinium-enhanced MR Angiography to detect pulmonary embolism showed a moderate test performance and modest patient compliance [PIOPED III (10)]. Meanwhile, novel 4D dynamic contrast-enhanced (DCE) lung perfusion magnetic resonance imaging (MRI) techniques (11) are widely available on current MRI systems and have shown excellent test performance in diagnosing CTEPH in a single center registry setting in Sheffield [ASPIRE registry (12)]. In addition, a non-contrast, free breathing ventilation perfusion MRI technique, known as the Fourier decomposition MRI method (13), has recently shown initial encouraging results in diagnosing chronic pulmonary embolism (14). In the CHANGE-MRI study we implemented phase-resolved functional lung (PREFUL) MRI as a secondary outcome parameter, which is a further development of Fourier Decomposition MRI depicting the whole breathing and cardiac cycle (15). These novel functional MRI techniques hold significant potential to be an equal rights non-ionizing alternative to VQ-SPECT in the near future, if they can demonstrate robust test performance in a prospective multicenter setting (Figure 2).

## Methods and Analysis

### Study Information

CHANGE-MRI is a prospective, multicenter, comparative phase III diagnostic study undertaken by the German Centre for Lung Research (DZL): Centre for Biomedical Research in Endstage and Obstructive Lung Disease Hannover (BREATH), the Universities of Giessen and Marburg Lung Centre (LGMLC), the Comprehensive Pneumology Centre Munich, the Translational Lung Research Centre Heidelberg (TLRC-H), the University of Sheffield, the Medical University of Vienna, the University Medical Centre of the Johannes Gutenberg University Mainz, the University Hospital Zurich and the University Hospital Regensburg. The study aims to demonstrate that functional lung MRI techniques can be an equal rights alternative to VQ-SPECT as a screening test in a diagnostic strategy for CTEPH where the positive findings of the screening test are verified with catheter pulmonary angiography or computed tomography pulmonary angiography (CPA/CTPA) in the diagnostic work-up.

Investigators from Hannover Medical School (MHH) designed the trial, and MHH acts as the study sponsor and is funded by the German Centre of Lung Research (DZL). A trial management group for the study comprises specialists from the disciplines of Pneumology, Nuclear Medicine, Radiology, Biostatistics, Medical Documentation and Medical Imaging and Computing. The study started recruitment in 2016. Ethical approval for the study was granted by MHH ethics committee (No 2678-2015). The trial is registered with ClinicalTrials.gov identifier *NCT02791282*.

### Objectives

The diagnostic strategy for CTEPH is basically composed of two components leading to a final diagnosis for or against CTEPH:

1. Presence of chronic pulmonary embolism, and
2. Presence of elevated pulmonary artery pressure.

The tests to be compared in this study (MRI vs. SPECT) are diagnostic interventions for chronic pulmonary embolism (chronic PE). Diagnosis of elevated pulmonary artery pressure follows the routine clinical assessment with right heart catheterization. As such, the study primarily deals with the first step diagnosis of chronic pulmonary embolism. The medical hypothesis of the CHANGE-MRI trial is that the diagnostic performance of novel functional lung MRI is sufficient to be an equal rights alternative to VQ-SPECT—the current clinical standard—in the detection of chronic PE in the diagnostic algorithm for CTEPH and thus can reduce the radiation burden for patients. Therefore, the MRI should identify almost all correct cases in the SPECT—strategy and should not increase the rate of false positive findings

The study will be considered successful in demonstrating that MRI can replace SPECT if the following co-primary hypotheses can be confirmed for the interrelation of MRI and SPECT:

- The probability that MRI is positive in patients who are SPECT positive and who have a positive gold standard is **larger than 95%** and,
- The probability that MRI is positive in patients who are SPECT negative and who have a negative gold standard is **smaller than 10%**.

To determine the sensitivity and specificity of functional MRI as co-primary endpoints of this diagnostic study, an alternative strategy would have been to send all study patients for a verification step with CPA/CTPA. However, this strategy was considered to imply an unacceptable increase in the diagnostic burden and radiation for patients who are not usually subjected to further diagnostic interventions.

Secondary objectives are:

- To demonstrate an additional benefit of MRI in comparison to SPECT with correct results of MRI (according to the gold standard) where SPECT showed false diagnoses
- To compare the performance of SPECT and MRI using conventional diagnostic measures: sensitivity, specificity, positive and negative predictive value
- to compare the performance of SPECT and MRI in subgroups of male and female and obese (BMI ≥ 28 kg/m^2^) and non-obese (BMI <28 kg/m^2^) patients
- to evaluate the inter-rater reliability in SPECT assessment, MRI-assessment and CPA/CTPA assessment between local in-place read and blinded read
- to compare SPECT and MRI regarding safety and procedure related limitations.

### Patients

The inclusion criteria are chosen to reflect exactly the clinical setting in the diagnostic algorithm for CTEPH. Here, MRI is positioned for an in-place validation compared to SPECT without any exclusion of subgroups compared to SPECT, except for inability to undergo MRI and pregnancy (see Table 1).

**Table 1:** Patient inclusion and exclusion criteria.

Inclusion	• Transthoracic echocardiography suggests pulmonary hypertension
	• Patients with clinical suspicion for CTEPH, scheduled for SPECT
	• Provided informed consent for the study
	• Age >18 years
Exclusion	• Inability to undergo MRI (e.g., due to claustrophobia, cardiac pacemaker, hypersensitivity to MR i.v. contrast imaging agents)
	• Women who are pregnant or breast feeding

### Diagnostic Methods

#### Index Test

**Figure 3:**
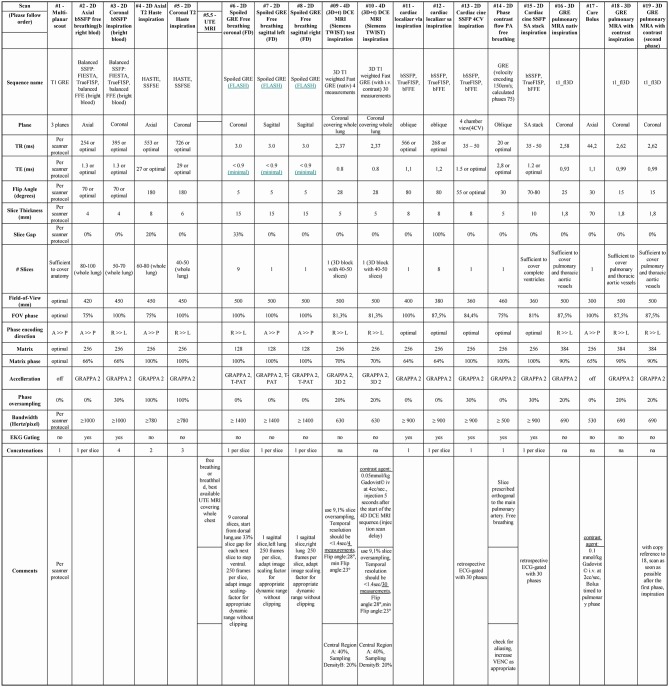
Detailed description of the MRI protocol.

A robust functional cardio-pulmonary-MRI exam that can be conducted within 30 min without radiation burden is used as novel index test. In brief, the MRI protocol consists of anatomical MR sequences, to depict thoracic pathology (ECG gated steady state free precession sequences and Half-Fourier Acquisition Single-shot Turbo spin Echo imaging covering the whole thorax both in axial and coronal planes), coronal 2D Fast low Angle Shot stacked time series for 50 sec each covering the whole thorax and 2 additional sagittal 2D Fast low Angle Shot planes (one for each lung) in free breathing for PREFUL MRI, contrast enhanced pulmonary MRA (4D time resolved gradient echo MRA and 3D pulmonary MRA, total gadolinium dose 1.5 mmol/kg) as well as cardiac function (short axis cine stack and 4 chamber cine view). While retrospectively ECG gated cardiac cine sequences are used in the study protocol, novel fast real-time cardiac cine sequences (i.e., compressed sensing MRI techniques) may be used, especially in cases of arrhythmia or shortness of breath (16). For detailed description of the MRI protocol see Figure 3. A central read for cardiac function and strain analysis is performed using dedicated cardiac software (CMR42, Circle Cardiovascular Imaging). For the primary read all MRI data is available except PREFUL MRI. In a sub-study all MRI data is available except 4D time resolved gradient echo MRA and 3D pulmonary MRA to test PREFUL as a secondary outcome parameter without the need for i.v. contrast for CTEPH diagnosis.

#### Reference Test

VQ-SPECT is used as the reference test in this study. In clinical practice, several CTEPH expert centers use perfusion single-photon emission computed tomography (Q-SPECT/CT) without concomitant ventilation SPECT (V-SPECT) to exclude or diagnose CTEPH, although not in compliance with the current ERS/ESC guidelines (8, 17). Therefore, in this study also Q-SPECT/CT is accepted as reference test, reflecting current clinical practice and real-world test performance. In this article, the reference test of VQ-SPECT or Q-SPECT/CT is termed SPECT. The duration of the intervention is ~60 min per patient. The radiation exposure applied by the reference test is ~1.2–2 mSv (17).

#### Diagnostic Gold Standard

Catheter pulmonary angiography or computed tomography pulmonary angiography (CPA/CTPA) is considered to be the current clinical gold standard for confirming chronic pulmonary embolism in a diagnostic algorithm (7). CTPA and CPA are well suited as verification step for the diagnosis of chronic pulmonary embolism (18). Nevertheless, they both have a considerable radiation burden on the patient. Therefore, in current diagnostic practice, in many expert centers only patients with a positive SPECT undergo a verification step with CPA/CTPA, whereas at some sites CPA/CTPA is used for verification of all patients. In this study, we only verify patients, if verification with CPA/CTPA is clinical routine at the respective study site. It is the responsibility of the treating pneumologist to decide whether verification by CPA/CTPA is clinically indicated. In any case, verification with CPA/CTPA is not part of study directives but a clinical decision.

A clinical follow-up after 6–12 months is implemented, where all patients are contacted and asked whether there has been any further intervention indicative (or contraindicative) of CTEPH, in order to identify cases diagnosed as false negative (or false positive, respectively) in the diagnostic work-up. Additionally, cases diagnosed as false negative by the screening test (SPECT negative patients are not verified by CPA/CTPA on a regular basis), but subsequently identified as positive can be captured by incorporation of the clinical follow up. Since the gold standard should capture the true diagnosis at inclusion, the clinical follow up diagnosis is restricted to be within 1 year of the inclusion to ensure that CTEPH—if diagnosed in the clinical follow up—was present at the inclusion already and did not develop afterwards.

With this trial design we avoid an increase in the diagnostic burden (i.e., the increased radiation of CPA/CTPA) for SPECT-negative patients without clinically indicated CPA/CTPA. A verification of all patients (to precisely estimate the sensitivity and specificity of functional MRI) was considered infeasible and ethically indefensible due to the application of the additional radiation dose required for CPA/CTPA without any clinical justification. Consequently, a composite gold standard for pulmonary embolism used in this trial combining SPECT, CPA/CTPA and follow-up. The composite gold standard is a diagnostic strategy with SPECT as screening test and CPA/CTPA as verification test, corrected by clinical diagnosis after 6–12 months:

- If CPA/CTPA is performed during the initial diagnostic work-up, the gold standard is set to the result of CPA/CTPA corrected for the clinical diagnosis after 6–12 months. In cases of mismatch, the clinical 6–12 months follow-up diagnosis overrules the CPA/CTPA diagnosis (e.g., if CPA/CTPA diagnosis is negative but 6–12 months diagnosis is positive, the gold standard diagnosis is positive).
- If CPA/CTPA has not been conducted during initial diagnostic work-up, the gold standard is set to the clinical diagnosis after 6–12 months.

#### Clinical Routine and Study Flow

**Figure 4:**
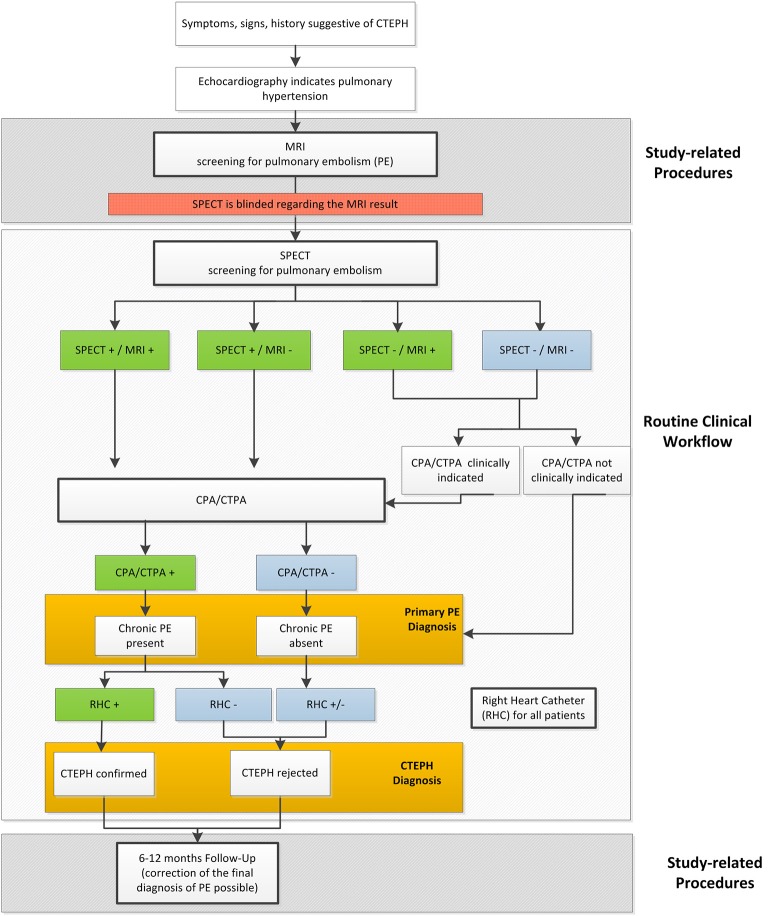
Integration of the MRI scan in the clinical workflow in the CHANGE-MRI study.

Figure 4 illustrates the current diagnostic work-up for pulmonary embolism in the diagnostic strategy for CTEPH, the additionally conducted study procedures and the path of verification and diagnostic decision in the CHANGE-MRI trial.

### In-place Assessment

In this study at each site standard operating procedures are implemented with the aim that the in-place assessment of SPECT (following the clinical routine) and MRI are performed and evaluated independently by two different readers from different teams. The respective diagnoses are termed in-place assessments and are used for secondary analyses. Both readers are aware of the medical history of the patient under investigation but are not allowed to know the diagnosis made with the aid of the other test, as the reference test is already clinically established and, as such, bares some information. In accordance, the system for electronic Case Report Forms is set up in a way that each reader‘s access is restricted to the respective imaging method.

If required by the treating pneumologist, CPA/CTPA is performed and evaluated at the study site, as well.

### Blinded-Reader Assessment

A centrally organized blinded reader assessment of SPECT and functional MRI will be implemented to ensure that no information carry-over occurs from clinical SPECT diagnosis to the functional MRI diagnosis or vice versa. Therefore, this blinded reader assessments will be used for the primary analysis. All SPECTs and functional MRIs will undergo a blinded read where independent expert readers are randomly selected for each center and each imaging method from one of the other centers. The second reader is not aware of the findings from the initial in-place read and the respective other imaging method but has access to the patient‘s clinical information used in the in-place SPECT read. Additional to ensuring a truly blinded assessment of the imaging methods, the standardized blinded reader assessment allows assessing inter-rater agreement and context sensitivity of the diagnostic evaluations. If there is a discrepancy between the in-place read and the second reader, a final consensus read will be performed by a third expert reader.

### Outcome Measures

SPECT and MRI will be compared based on a standardized blinded reader assessment of the imaging methods as well as based on the in-place assessments as part of a workflow incorporated into the clinical routine. To definitely exclude information carry-over in the primary analysis, the following co-primary endpoints are calculated based on the standardized blinded reader assessment:

- The proportion of patients with positive MRI in the group of patients who have a positive SPECT diagnosis for chronic pulmonary embolism and who are positive in the gold standard
- The proportion of patients with positive MRI in the group of patients with negative SPECT who are negative in the gold standard.

The co-primary endpoint will also be analyzed based on the in-place assessment and results will be compared to assess the inter-rater agreement and context sensitivity of the diagnostic evaluations and to evaluate the external validity and generalizability of the primary results. Additional secondary endpoints based on the in-place assessment and the blinded-reader assessment include the diagnostic measures sensitivity, specificity, positive and negative predictive values of MRI and SPECT as compared to the defined gold standard. To assess additional benefit of the MRI compared to the SPECT, the proportion of patients with positive MRI in the group of patients who have a negative SPECT, but are positive in the gold standard, and the proportion of patients with negative MRI in patients who are SPECT positive, but have a negative gold standard, will be evaluated.

To assess the safety, the occurrence of allergic reactions or other adverse events, non-completed procedures, reasons for non-completion, non-diagnostic procedures, reasons for non-diagnostic, and the quality of the images will be evaluated for both imaging techniques.

### Sample Size

The sample-size calculation was based on a substudy from the Sheffield ASPIRE registry with 132 patients suspected for CTEPH (12). The prevalence of CTEPH was around 60% here, sensitivities and specificities were 96 and 90% for Q-SPECT and 97 and 92% for functional MRI. It was not appropriate to directly utilize these numbers for sample size estimation: First, the primary analysis of the CHANGE-MRI trial uses different, unconventional endpoints due to the incomplete verification by CPA/CTPA. Second, it was unclear, in how far unverified (by CPA/CTPA) patients will occur in the study setting and how large the resulting differential verification bias might be.

Thus, a simulation study was conducted based on the ASPIRE study simulating 1,000 patients in different scenarios. Estimates for the primary endpoint proportions were taken from the mean diagnostic table of 5,000 simulation runs (see Table 2): the proportions of patients with positive MRI within those with positive SPECT and positive gold standard was estimated to be 98% and the proportion of patients with positive MRI within those with negative SPECT and negative gold standard was estimated to be 6%. These estimates were used for sample size calculations with a Chi^2^-test. For both co-primary hypotheses, a one-sided type-I-error of 2.5% and a power of 80% were used. Sample size calculations for the individual hypothesis resulted in 331 patients with positively verified SPECT and 388 patients with negative SPECT and negative gold standard. To calculate the expected overall sample size to achieve this sample sizes for the subgroups, the expected diagnostic table was calculated based on the simulation study. For each hypothesis, the expected overall sample size was calculated taking into account the expected prevalence of each subgroup (58 and 36%, respectively, see Table 2). Consequently, the final sample size was determined by the higher sample size, which is associated with the second primary objective [Table 2, part (b)]. A total of *N* = 1080 required patients are anticipated.

**Table 2:** Sample size calculation based on a simulation study: **(A)** Expected diagnostic table and **(B)** expected required sample sizes for the co-primary endpoints.

**(A) Expected diagnostic table**						
		**Gold standard**				
		**+**		**–**		
		**SPECT**		**SPECT**		
		**+**	**–**	**+**	**–**	
MRI	+	562 (98%)^*^	20 (82%)^*^	11 (28%)^*^	21 (6%)^*^	
	–	14 (2%)^*^	4 (18%)^*^	29 (72%)^*^	339 (94%)^*^	
**Sum**		576 (58%)^#^	24 (2%)^#^	40 (4%)^#^	360 (36%)^#^	1000
**(B) Expected required sample sizes for co-primary endpoints**						
(1) Proportion of patients with MRI+ if gold standard+ and SPECT+		Expected: 98%	Targeted: Min 95%	Required patients gold standard+ and SPECT+: 331	Expected total number required: 571	
(2) Proportion of patients with MRI+ if gold standard– and SPECT–		Expected: 6%	Targeted: Max 10%	Required patients gold standard– and SPECT–: 388	Expected total number required: 1080	

* *Column percentages*.

# *Row percentages*.

### Statistical Analysis

The primary analysis will be performed in a modified intention-to-treat population, i.e., patients will only be excluded from the analysis, if for all three diagnostic tests no assessment (neither in-place nor blinded) is available and no further follow-up data on the clinical diagnosis after 6–12 months is available. The overall two-sided type-I-error probability is set to 5%. Point estimates and 95% Wilson confidence intervals will be calculated for both co-primary endpoints based on the blinded reader assessments. Since both endpoints are evaluated co-primarily, no correction for the overall type-I-error probability is necessary.

Imputation of missing values is conducted dependent on whether any assessments of SPECT, MRI or CPA/CTPA images and clinical follow up diagnosis are available or not. If either in-place or blinded reader assessments are available, missing values of MRI or SPECT will preferably be replaced by the results of the respective available assessments. In the case of missing values because the test has not been performed at all or neither in-place nor blinded assessment are available, missing values of MRI or SPECT will be replaced using a conservative strategy in favor of SPECT, where missing information for the experimental functional MRI in all instances is counted such that sensitivity and specificity are diminished as compared with SPECT.

If missing values occur for all three diagnostics MRI, VQ-SPECT, and gold standard (e.g., because the patient decided against the study directly after written informed consent) and no further follow-up information is available for the patient, the patient is omitted from the analysis population and no imputation will be performed.

Secondary analyses will be performed in line with primary analyses using point estimates and 95% Wilson confidence intervals for the various proportions of interest. Safety endpoints will be evaluated descriptively with absolute and relative frequencies of complications and malfunctions of MRI and SPECT and will be compared using risk differences and the Chi^2^-tests. As sensitivity analyses, the primary analysis will be repeated in the per-protocol population based on the blinded reader assessment, comprising all patients without missing co-primary endpoints. Additionally, a sensitivity analysis will be conducted in the per-protocol population based on the in-place assessment, comprising all patients, where in-place MRI diagnosis has been entered into the eCRF before SPECT assessment so that the blinding can be considered to be intact.

### Methods Against Bias

All centers document the age and gender of all patients with suspected CTEPH undergoing SPECT in a screening log. There is agreement that information will be made available in all centers from the hospital administration to assess the degree of representativeness of the investigated patient population for the overall CTEPH-population.

It is the primary aim of this diagnostic study to demonstrate perfect agreement between SPECT and functional MRI in all cases, where the SPECT can be considered to be correct. Therefore, it is of utmost importance that the two diagnostic tests are conducted and evaluated independently. To ensure internal validity of the co-primary endpoint, the diagnoses used in the primary analysis are determined in a standardized blinded reader assessment of the SPECT and MRI images.

Standard operating procedures are developed for each study site in order to achieve that the in-place assessment of MRI and SPECT is performed independently and blinded against each other.

Before commencement of the CHANGE-MRI study at the respective centers, local readers are trained on the basis of ~15 MRI test cases that are provided by the centers to exclude effects of learning curves on the outcome measures.

## Discussion

In this study also Q-SPECT/CT is accepted as an approximation of the VQ-SPECT as reference test, reflecting current clinical practice and real-world test performance, although not in compliance with the current ESC/ERS guidelines (8, 17). This omission of V-SPECT will affect in particular specificity of the reference test in cases of Q-SPECT/CT, for CT cannot be regarded as a fully equivalent substitute for V SPECT.

For primary evaluation, the blinded reader assessment of SPECT and MRI are used to ensure the independency of the assessments and maximize internal validity. On the other hand, external validity of the results might be reduced by the standardized blinded assessment, which does not necessarily reproduce the clinical field of application completely. Consequently, for analyzing the external validity and generalizability of the results based on the primary blinded assessment, the in-place assessments of SPECT and MRI are used as secondary endpoints. While the independence of these assessments cannot be guaranteed, the clinical setting is well represented.

To avoid an increased radiation burden, in this trial the current gold-standard instrument CPA/CTPA is routinely only applied in SPECT-positive patients, which might induce differential verification bias (6, 19, 20). To mitigate this limitation, the follow-up information up to 1 year after study inclusion will be included into the final diagnosis of PE by correcting negative cases, which subsequently are identified as positive in the follow-up period or (although less probable) correcting positive cases, which subsequently are identified as negative.

Ethics votes from the Institutional Review Boards at all participating centers were obtained. The CHANGE-MRI study is designed so that no extra radiation in addition to the clinically indicated radiation-based imaging tests is applied to the study participants. Thus, approval by the Federal Office for radiation protection (Bundesamt für Strahlenschutz) was not required.

This study is conducted in compliance with the Declaration of Helsinki, ICH-E6-guidance, and for design issues regarding diagnostic validation studies, and the EMA Guideline on the Clinical Evaluation of Diagnostic Agents.

The multicenter CHANGE-MRI study could pave the way for lung MRI methods to be an equal rights alternative to VQ-SPECT in the diagnostic pathway for CTEPH. By generating robust evidence both from in-place assessments and a standardized blinded reader assessment, this trial will generate the data needed for a thorough comparison of SPECT and MRI technology.

## Author Contributions

JV-C, AKo, FL, AKa, and TD contributed the conception and design of the study. FL and JV-C drafted the manuscript. AV contributed image editing algorithms. TD, TA, MMH, HG, FRo, SH, DS, GK, HAG, FRe, CH, EG, DB, MH, IL, JB, PB, JD, K-HS, K-FK, TF, SU, OH, MP, CJ, DK, AS, JW, and JV-C are contributing to the acquisition of data. All authors critically revised the manuscript and approved the submitted version.

### Conflict of Interest

The authors declare that the research was conducted in the absence of any commercial or financial relationships that could be construed as a potential conflict of interest.

## Abbreviations

BREATH: Biomedical Research in Endstage and Obstructive Lung Disease Hannover
CPA/CTPA: catheter pulmonary angiography or computed tomography pulmonary angiography
CTEPH: thromboembolic pulmonary hypertension
DCE: dynamic contrast-enhanced
MHH: Hannover Medical School
MRI: magnetic resonance imaging
LGMLC: Universities of Giessen and Marburg Lung Center
PE: pulmonary embolism
Q-SPECT/CT: perfusion single-photon emission computed tomography
TLRC-H: Comprehensive Pneumology Center Munich and the Translational Lung Research Center Heidelberg
USFD: University of Sheffield
V-SPECT: ventilation single-photon emission computed tomography
VQ-SPECT: ventilation–perfusion single-photon emission computed tomography.
